# Pericardial effusion in an Indian context: clinical insights and dynamics from a tertiary care centre

**DOI:** 10.1186/s12872-024-04381-1

**Published:** 2024-12-19

**Authors:** Nafeez Javed Shaik, Sindhu Sujeeth Hegde, Shubha Seshadri, Mounika Cherukuri

**Affiliations:** 1 Department of General Medicine, Kasturba Medical College, Manipal, Manipal Academy of Higher Education, Manipal, 576104 Karnataka India; 2 Faculty of General Medicine, Department of General Medicine, Kasturba Medical College, Manipal, Manipal Academy of Higher Education, Manipal, 576104 Karnataka India; 3 Kasturba Medical College, Manipal, Manipal Academy of Higher Education, Manipal, 576104 Karnataka India

**Keywords:** Pericardial effusion, Clinical features, Cardiac tamponade, Constrictive pericarditis, Etiology, India

## Abstract

**Background:**

Pericardial effusion (PE) indicates the build-up of fluid within the pericardial sac, which encases the heart. The present study was undertaken to assess the clinical profile, etiology of pericardial effusion and to determine the correlation of cardiac tamponade and constrictive pericarditis with etiology.

**Methods:**

A prospective observational hospital based longitudinal study was undertaken among the 88 newly diagnosed and known patients of pericardial effusion who are above 18 years. The clinical profile of pericardial effusion including history, examination, standard lab parameters routinely done including thyroid function tests, HIV Serology, ECG, Echocardiography and imaging if done (HRCT thorax), pericardial fluid analysis (if performed) were elicited.

**Results:**

Majority of the patients were males (55.7%), with a mean age of 51.3 years**.** Among the 88 patients of pericardial effusion, 20 had cardiac tamponade, 13 individuals were diagnosed with constrictive pericarditis. Dyspnea was the most common presenting complaint (65.9%). Chronic kidney disease / uremia is the most common cause of pericardial effusion accounting for 25%, followed by neoplastic (20.5%) and tuberculosis (17%). While in cardiac tamponade patients neoplasm followed by tuberculosis were the most common etiology, patients with constrictive pericarditis had tuberculosis followed by chronic kidney disease as the most common etiology. Echocardiography features were not significantly different according to the etiology of the pericardial effusion (*p* > 0.05). Thickened pericardium found in the echocardiography showed maximum specificity (76.9%), while thickened fluid/exudates showed maximum sensitivity (65.2%) and negative predictive value (77.1%) for tuberculous pericardial effusion.

**Conclusion:**

Chronic kidney disease, closely followed by infections (mostly tuberculosis), are the frequent causes of PE in the present settings. Breathlessness is the most frequent clinical feature in the patients of PE. Fibrin strands, thickened pericardium, thickened fluid in Echocardiography assists in diagnosing tubercular pericardial effusion. Cardiomegaly in chest X-ray or CT scans should further prompt towards diagnosing pericardial effusion. It is essential to incorporate these findings into the clinical practice, by evaluating the patients presenting with breathlessness for PE. CKD needs to be placed on par with tuberculosis while suspecting the etiology of the PE in the present settings. ADA levels in pericardial fluid (> 40) can be considered as a specific marker for tubercular PE.

## Background

A pericardial effusion (PE) indicates the build-up of fluid within the pericardial sac, which encases the heart. This sac is composed of 2 layers. The outer layer, known as the fibrous parietal pericardium, is thicker and made of collagen and elastin; it is connected to the lungs, diaphragm, sternum, great vessels, and other surrounding mediastinal structures. The inner layer, called the visceral pericardium, is a thin, single-cell layer that directly adheres to the surface of the heart (cardiac epicardium). The amount of serous fluid in a healthy person's pericardial sac ranges from 15 to 50 ml [1, 2]. If not identified and treated promptly, the condition may cause serious cardiovascular compromise, which may eventually end in cardiac tamponade and death [3]. The development of cardiac tamponade and collapse of the circulatory system is the primary clinical importance of pericardial effusion. Tamponade is very frequent in individuals with hemorrhagic effusions, neoplastic involvement, and less commonly seen with infections related to bacteria, fungi, and HIV [4]. In developed environments, idiopathic PE accounts for half of cases; iatrogenic, cancerous, infectious, and inflammatory etiologies account for the other instances [5, 6]. PE mostly originates from infectious diseases in underdeveloped nations, including tuberculosis (TB) and bacterial etiologies such as staphylococcus [7, 8].

Common signs of PE include orthopnoea, dyspnoea with exercise, chest discomfort and fever. In a patient without any cardiovascular abnormalities, the physical examination might be normal. Prominent symptoms of cardiac tamponade include tachycardia, elevated “jugular venous pressure (JVP)”, muffled and distant heart sounds, hypotension, and pulsus paradoxus [3]. Echocardiography (ECHO) may facilitate the diagnosis of PE. Based on size, PE is categorized as mild which is less than ten mm, moderate that is between ten and twenty mm while severe had more than twenty mm on echocardiograms [3]. ECHO also aids in determining whether cardiac tamponade is present or absent. Echocardiographic features of cardiac tamponade include plethora in the “inferior vena cava (IVC)”, collapse of the free wall of the “right atrium (RA)” during systole, collapse of the free wall of the right ventricle during diastole, and exaggerated changes in the respiratory cycle in the “atrioventricular (AV)” valves. Assays for chemistry, cytology, and microbiology may be performed on pericardial fluid [9]. Pericardiocentesis should be performed on any patient who has cardiac tamponade and is suspected of having bacterial or neoplastic etiology. Once the patient is clinically stabilised, further therapeutic options are chosen in accordance with the PE's underlying etiology. For traumatic PE, pericardiocentesis or an catheter insitu are employed; for non-traumatic PE, sternotomy or pericardial window are recommended [9, 10]. Timely pericardiocentesis or pericardial drainage procedures have been shown to be safe and was found to be correlated with a lower death rate in patients with hemodynamically compromised PE [11].

It is vital to understand the etiopathology of PE as well its correlation with the complications for better management, in the respective settings. The present study was undertaken to assess the clinical profile, etiology of pericardial effusion and to determine the correlation of cardiac tamponade and constrictive pericarditis with their etiology.

## Materials and Methods

A prospective observational hospital based longitudinal study was undertaken among the newly diagnosed and known patients of pericardial effusion who are above 18 years, and on follow up at Kasturba Hospital, Manipal during the study period. Patients who came for trivial pericardial effusion traumatic cases (RTA, chest trauma), postCardio thoracic surgeries related were excluded. Traumatic effusions were excluded since they are due to surgical reasons or injuries rather than infections or malignancies. This exclusion ensured certain degree of homogeneity in our study population. Complete enumeration of all patients who were eligible from April 2023 to August 2024 were included in the study.

### Methodology

The patient details noted were demographic characteristics (age, gender), symptoms, signs, general physical examination, particularly of the cardio-vascular system. The clinical profile of Pericardial effusion including history, examination, standard lab parameters including thyroid function tests, HIV Serology, ECH, ECHO and imaging (HRCT thorax) were done. Patients reporting fever symptoms have been observed in the hospital for elevated body temperature, with those exceeding 99 degrees Fahrenheit have been taken as febrile. Patients with documented previous weights in hospital records have been compared to their current weight, whereas for those without prior documentation, weight loss is noted based on the history given by the patient. All the tests and radiological investigations done were part of standard care routinely done in outpatient or inpatient. No additional tests were done for the purpose of research.

#### Echocardiography

Because of its real-time imaging capabilities, portability, and capacity to evaluate hemodynamic impact, echocardiography is the main imaging modality used to diagnose pericardial effusion. When the effusion volume is greater than 50 mL, an anechoic (dark) separation of pericardial layers may be a sign that fluid is present. The posterior region, close to the left atrium and descending aorta, is usually where this separation is first observed in a supine patient [9].

I.  < 5 mm depth: 50–100 mL (mild effusion) is the approximate volume.
II. 5–10 mm depth: Mild to moderate volume range of 100–250 mL.
III. Depth of 10–20 mm: Moderate to large volume range of 250–500 mL.
IV.  > 20 mm depth: Severe effusion with a volume greater than 500 mL.

Effusions are frequently categorized according to depth for simplifying it:

I.  < 10 mm: Tiny.
II. 10–20 mm: Moderate.
III.  > 20 mm: Huge.

Preferred Views: The most popular views for a comprehensive examination are the parasternal long-axis and subcostal four-chamber views, which aid in distinguishing pericardial effusion from pleural effusions and epicardial fat.

#### Imaging with CT

By adding more structural detail and evaluating any surrounding anatomy for pathological changes, CT scans can be used in conjunction with echocardiography.

Normal versus Abnormal Thickness: A CT scan shows that the pericardium is normally up to 2 mm thick. In most cases, if the pericardium appears thicker than 3–4 mm, diffusion is deemed abnormal.

Incidental Finding: Minor effusions in hospitalized patients could be discovered by chance. Nonetheless, CT is useful for determining fluid density, which may indicate the cause of the effusion, and for excluding masses or tumors that are compressing the pericardium.

Fluid Localization: By displaying distinct borders and anatomical relationships, CT aids in distinguishing pericardial effusion from other chest pathologies, including pleural effusions and fat pads.

#### Analysis of fluids

Etiological determination requires pericardial fluid analysis, which is usually carried out following fluid aspiration.

Transudate vs. Exudate: To determine whether the fluid is an exudate (indicating an infection, cancer, or autoimmune cause) or a transudate (due to heart failure), it is examined for protein, LDH, and cellular composition.

Specific tests: In cases of suspected infection or cancer, additional tests such as cytology, bacterial culture, and PCR for tuberculosis can help identify the underlying cause.

These methods, with combined fluid analysis, CT imaging, and echocardiography, enables a nuanced evaluation of pericardial effusions and directs the proper management according to their severity and etiology [9].

### Ethical considerations

Before beginning the research, approval from institutional ethics committee of the Kasturba Medical College and Kasturba Hospital, dated January 10, 2023, was obtained. All patients who participated in the research gave their written informed consent. At every stage, the protection of patient privacy and data secrecy was ensured.

### Statistical analysis

Statistical analysis was performed on all the data after it was first collated and recorded on a sheet of Microsoft Excel, and then transferred to the SPSS program. Frequency and percentage were used to represent qualitative data that was collected. For continuous variables, the data is provided as the mean and standard deviation. Sensitivity, specificity, positive predictive value and negative predictive value of the echocardiographic manifestations and ADA levels of tuberculous pericardial effusion was calculated. Chi-square test was applied to test association between echocardiographic features and the etiology, since both are categorical variables, and a p value < 0.05 was considered as significant.

## Results

Among the 88 patients of pericardial effusion, 55.7%, (49) individuals had moderate pericardial effusion accounting for the majority of population studied, followed by 39.8% with severe pericardial effusion. Among them, 20 had cardiac tamponade and 13 individuals were diagnosed with constrictive pericarditis. Majority of the patients were males (55.7%) and the mean (SD) age of the patients was 51.3 (19.1) years. Dyspnea was the most common presenting complaint (65.9%). Other demographic and clinical features of the patients with pericardial effusion in the current study are enumerated in the Table 1.

**Table 1:** Demographic and clinical features of the patients with pericardial effusion (N = 88)

	**Frequency**	**Percentage**
**Sex**		
Male	49	55.7
Female	39	44.3
**Age (Years)**		
18–30	12	13.6
31–40	8	9.1
41–50	22	25
51–60	22	25
61–70	14	15.9
71–80	7	8
81 +	3	3.4
**Symptoms**		
Dyspnea	58	65.9
Swelling of limbs	36	40.9
Chest pain	31	35.2
Fever	25	28.4
Abdominal distension	22	25
Loss of weight	21	23.9
Arthralgia	10	11.4
Skin rashes	7	8
**Cardiovascular System signs**		
Raised JVP	65	73.9
Muffled heart sounds	50	56.8
Kussmaul’s sign	46	52.3
Pericardial rub	41	46.6
Pulsus paradoxus	31	35.2
**ECG**		
Low voltage complexes	45	51.1
Electrical alternans	7	8
Atrial fibrillation	4	4.5
T wave inversions	2	2.3
Anterior wall STEMI	1	1.1
Left bundle branch block	1	1.1
Right ventricular hypertrophy	1	1.1
**Chest X-ray**		
Normal	37	42.1
Cardiomegaly	26	29.6
Consolidation	13	14.8
Pleural effusion	11	12.4
Pulmonary edema	1	1.1
**General examination**		
Pallor	56	63.6
Pedal edema	43	48.9
Clubbing	18	20.5
Lymphadenopathy	12	12.6
Icterus	3	3.4

*JVP* jugular venous pressure, *STEMI* ST Elevation Myocardial Infarction, *ECG* electrocardiogram

The descriptive statistics of the vitals and biochemical parameters of the patients with pericardial effusion are enumerated in Table 2.

**Table 2:** Descriptive statistics of the vitals and biochemical parameters of the patients

	Mean	Median	Std. Deviation	IQR
PR (bpm)	90.2	89	18.5	80,104
SBP(mm of Hg)	116.8	120	21.1	108.5,120
DBP(mm of Hg)	76.7	80	11.5	80,80
RR(cpm)	24.6	24	3.2	22,27.5
HB(g/dl)	10.7	10.5	2.4	9.4,11.8
Platelet count(cells/cmm)	287,729.9	263,000	171,932.2	173,000,344,000
WBC count(cells/cmm)	12,309.3	9550	20,332.3	6950,12,550
Urea(mg/dl)	44.5	28.5	39.5	18,56.8
Creatinine(mg/dl)	3.1	0.9	10	0.7,2.4
Na (meq/dl)	132.5	135	14.2	131,137
K meq/dl)	4.5	4.4	0.9	3.9,4.8
TSH(mIU/l)	5.9	2.8	16	1.7,4.3
ESR(mm/hr)	38.1	26.0	30.7	15,58

*PR* pulse rate, *SBP* systolic blood pressure, *DBP* diastolic blood pressure, *RR* respiratory rate, *HB* hemoglobin, *WBC* white blood cells, *Na* sodium, *K* potassium, *TSH* thyroid stimulating hormone, *ESR* erythrocyte sedimentation rate, *IQR* interquartile range

**Fig. 1:**
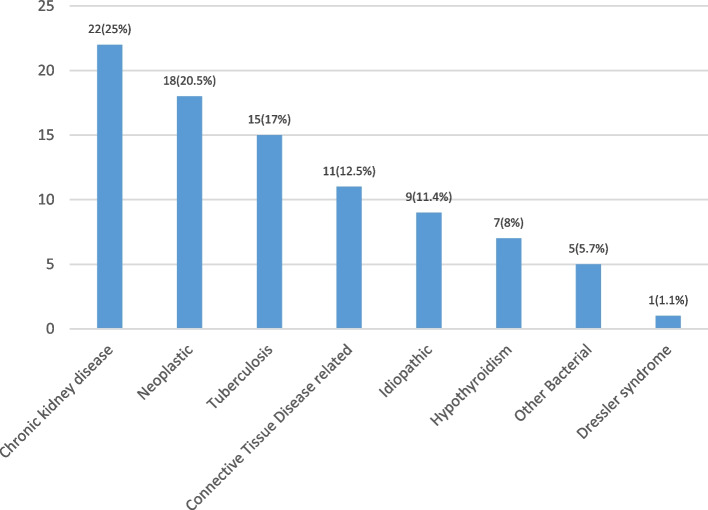
Etiological factors for pericardial effusion

**Fig. 2:**
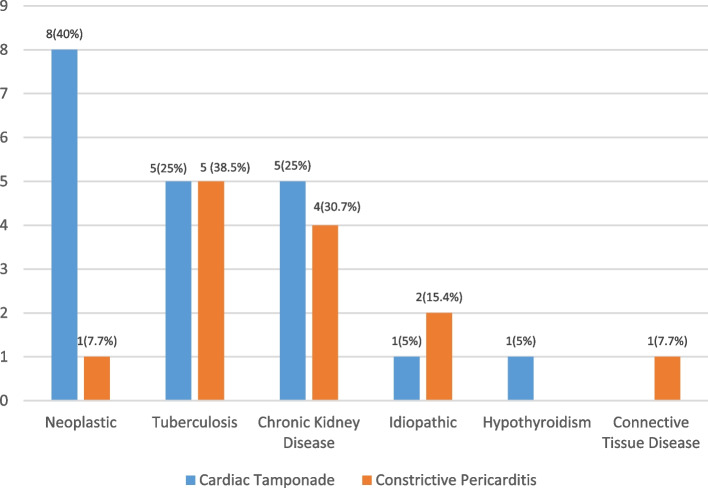
Etiological factors for Pericardial effusion: Cardiac Tampanode vs Constrictive Pericarditis

Chronic kidney disease (CKD) / uremia is the most common cause of pericardial effusion accounting for 25%, followed by neoplastic (20.5%) and tuberculosis (17%). Connective tissue disease-related conditions account for 12.5%, and idiopathic cases are 10.2% (Fig. 1). While in cardiac tamponade patients, neoplastic followed by chronic kidney disease/uremia were the most common etiology, patients with constrictive pericarditis had tuberculosis followed by chronic kidney disease as the most common etiology. (Table 3, Fig. 2). However, no statistically significant difference in the distribution of etiologies between cardiac tamponade and constrictive pericarditis (*p* = 0.459) was noted.

**Table 3:** Etiological factors for Pericardial effusion

	**Frequency**	**Percentage**
**Pericardial effusion**		
Chronic kidney disease	22	25
Neoplastic	18	20.5
Tuberculosis	15	17
Connective Tissue Disease related	11	12.5
Idiopathic	9	11.4
Hypothyroidism	7	8
Other Bacterial	5	5.7
Dressler syndrome	1	1.1
**Cardiac Tamponade**		
Neoplastic	8	40
Tuberculosis	5	25
Chronic Kidney Disease	5	25
Idiopathic	1	5
Hypothyroidism	1	5
Connective Tissue Disease	0	0
**Constrictive Pericarditis**		
Tuberculosis	5	38.5
Chronic Kidney Disease	4	30.7
Idiopathic	2	15.4
Neoplastic	1	7.7
Connective Tissue Disease	1	7.7
Hypothyroidism	0	0

**Fig. 3:**
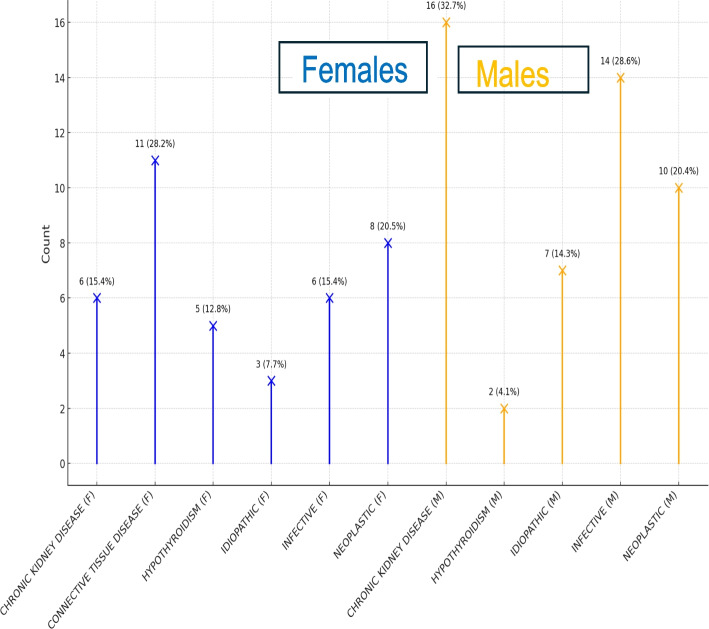
Gender wise etiology of the study patients

Among females, connective tissue disease was the most common cause (28.2%), while among males it was chronic kidney disease (32.7%) (Fig. 3).

**Fig. 4:**
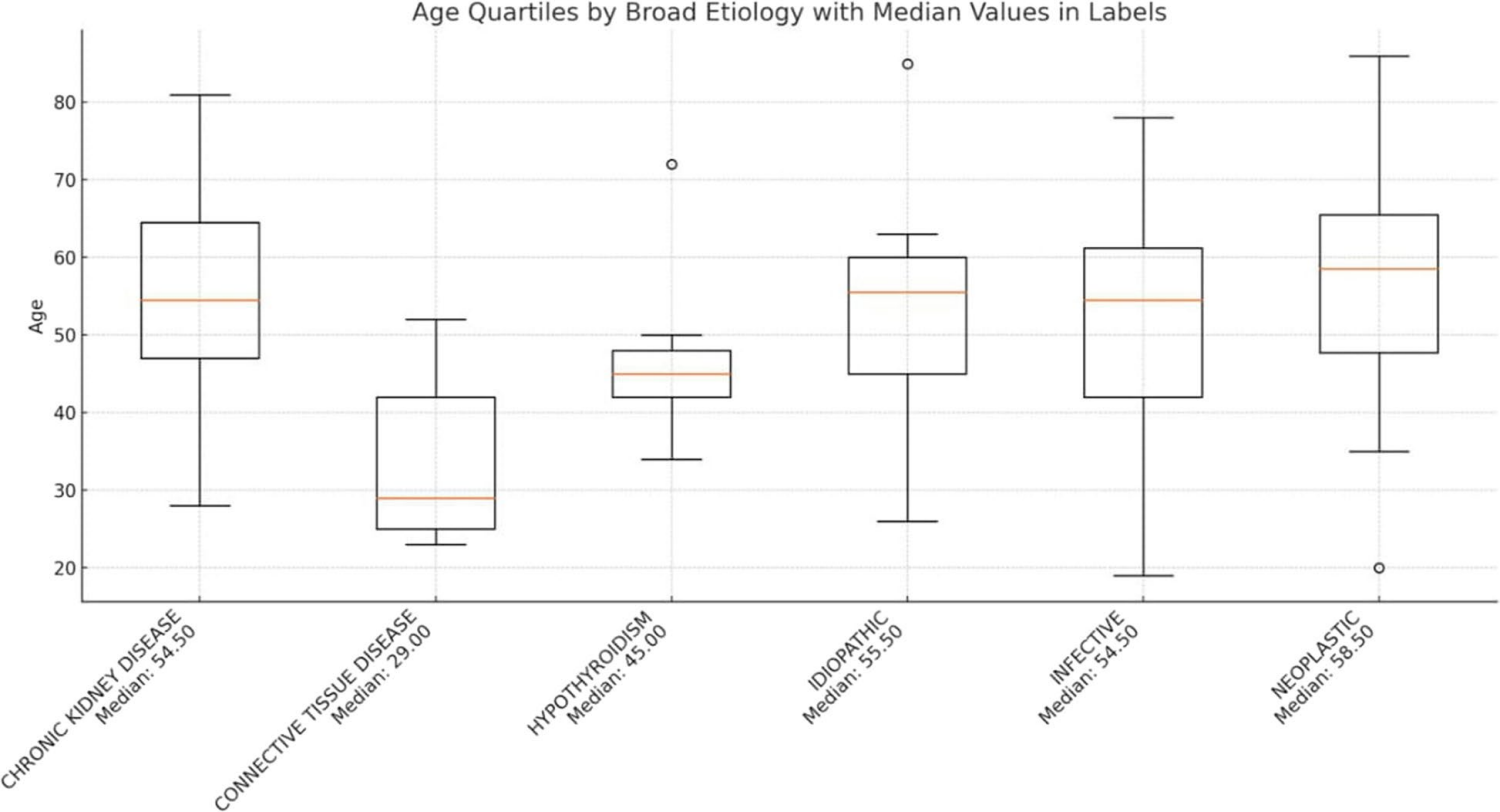
Age quartiles of the etiology of the study patients

Median age of the patients who had neoplastic etiology was the highest (median = 58.5 years) than the patients with other etiologies. Patients with connective tissue disorders had the lowest age (median = 29 years) (Fig. 4).

**Fig. 5:**
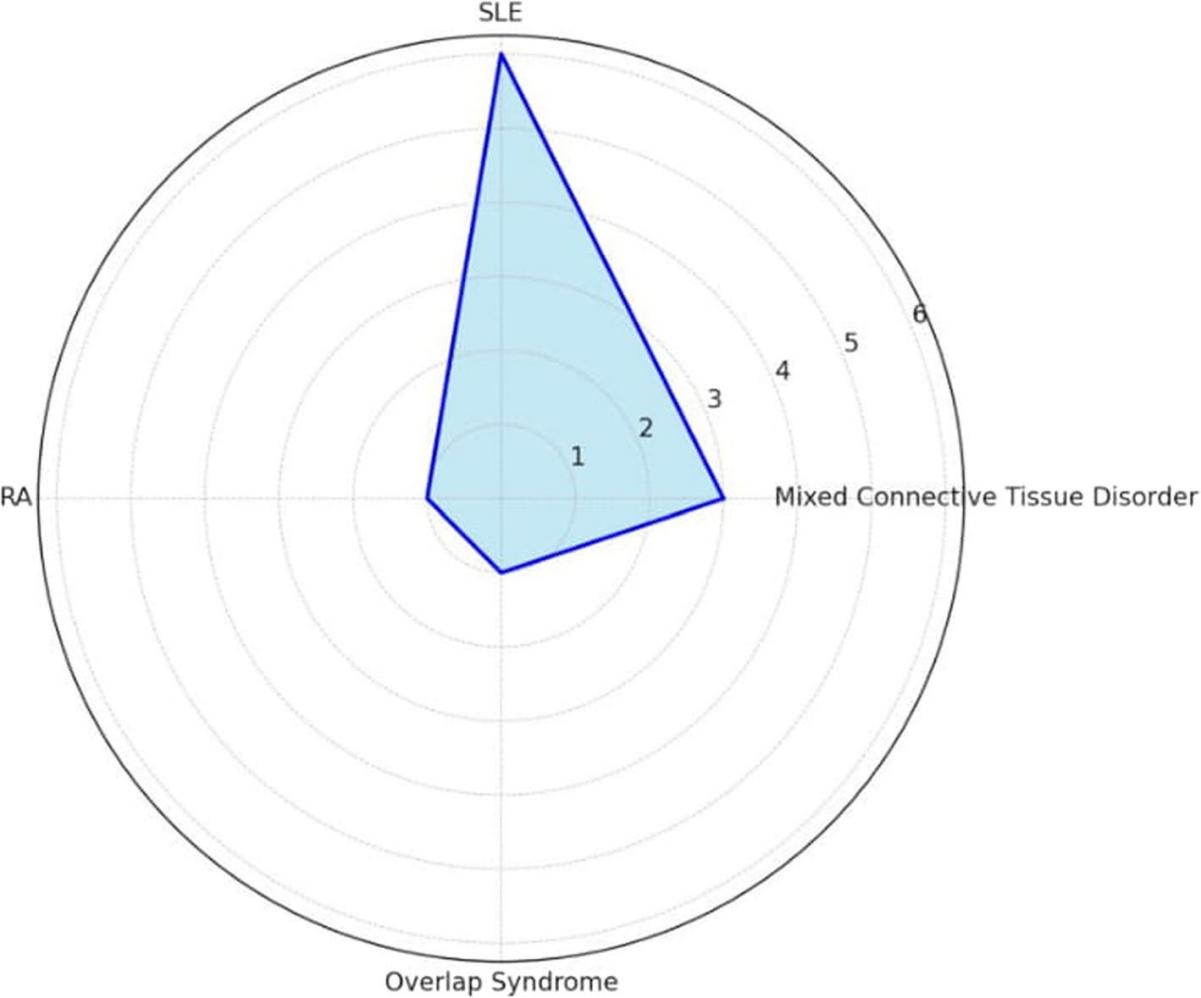
Radar plot showing distribution among connective tissue diseases

SLE was the most common connective tissue disease noted among the patients in the present study (Fig. 5).

Echocardiography features were not significantly different according to the etiology of the pericardial effusion (*p* > 0.05) (Table 4).

**Table 4:** Etiology versus Echo characteristics among the study patients

Echo feature	Tuberculous			Malignant			Idiopathic		
	Nos	%	*p*-value	Nos	%	*p*-value	Nos	%	*p*-value
Fibrin strands	14	56	0.461	3	12	0.221	2	8	0.317
Thickened pericardium	10	52.7	0.67	1	5.3	0.095	2	10.5	0.551
Thickened fluid	15	55.6	0.464	3	11.1	0.172	2	7.4	0.264

Thickened pericardium found in the echocardiography showed maximum specificity (76.9%), while thickened fluid/exudates showed maximum sensitivity (65.2%) and negative predictive value (77.1%) for tuberculous pericardial effusion. (Table 5).

**Table 5:** Diagnostic validity of echocardiographic manifestations of tuberculous pericardial effusion

Echo Features	Sensitivity (%)	Specificity (%)	Positive predictive value (%)	Negative predictive value (%)
Fibrin strand	60.9	71.8	56.0	75.7
Thickened Fluid/Exudates	65.2	69.2	55.6	77.1
Thickened pericardium	43.5	76.9	52.6	69.8

ADA levels of ≥ 20 U/L had a sensitivity and specificity of 53.9% and 75%, respectively. Lymphocyte/Neutrophil Ratio > 1 also had a sensitivity and specificity of 53.9% and 75% respectively. (Table 6).

**Table 6:** Diagnostic validity of ADA levels in pericardial effusion for tuberculosis

Echo Features	Sensitivity (%)	Specificity (%)	Positive predictive value (%)	Negative predictive value (%)
ADA level ≥ 40 U/L	30.8	100	100	30.8
ADA level ≥ 30 U/L	38.5	75	83.3	27.3
ADA level ≥ 20 U/L	53.9	75	87.5	33.3
Lymphocyte/Neutrophil Ratio > 1	53.9	75	87.5	33.3

*ADA* adenosine deaminase

**Fig. 6:**
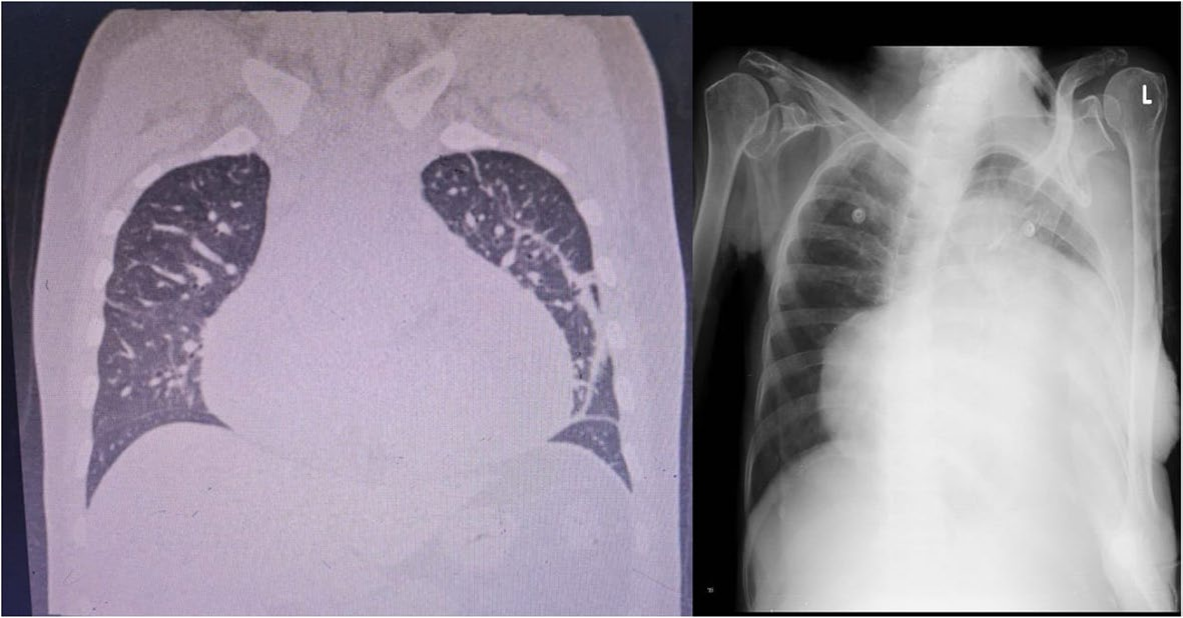
HRCT & Chest X-Ray showing gross cardiomegaly

Gross cardiomegaly has been observed in the HRCT and chest X-ray among a patient in the study (Fig. 6) A typical chest X-ray and HRCT (High-Resolution Computed Tomography) imaging are shown in this figure, both of which demonstrate considerable pericardial effusion and substantial cardiomegaly. The HRCT scan offers a high-resolution image that makes it possible to examine the heart, lungs, and surrounding structures in detail. A substantial pericardial effusion with a maximal thickness of 3.9 cm is revealed by HRCT in this study, which helps explain the the enlarged cardiac silhouette taking up a sizable amount of the thoracic cavity. Furthermore, atelectatic bands are observed in the right middle lobe, the anterior and lingular segments of the left upper lobe, and the basal regions of the left lower lobe exhibit subsegmental atelectasis and a slight left pleural effusion. In attenuation, the trachea and major bronchi appear normal, as does the remainder of the lung parenchyma. Sections of the thyroid gland, liver, and spleen show no localized abnormalities, and the scan's soft tissues and bones appear normal. A brief overview is given by the chest X-ray, which reveals an enlarged cardiac shadow with a "globular" or "water-bottle" shape that is characteristic of substantial cardiomegaly and suggests pericardial effusion. While the HRCT provides thorough anatomical insights into the size, shape, and surrounding structures of the heart, the chest X-ray is the primary screening method. These imaging techniques together provide clues to physicians to help diagnose and treat pericardial effusion and related problems by improving the assessment of consequences such as compression of nearby structures.

**Fig. 7:**
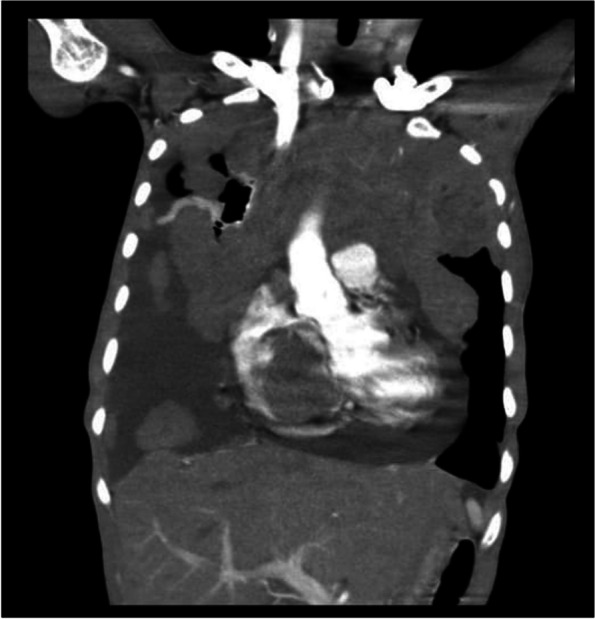
Anterior mediastinal mass with pleural metastasis and moderate pericardial effusion – Thymolymphoma

Figure 7 shows the anterior mediastinal mass with pleural metastasis and moderate pericardial effusion.

**Fig. 8:**
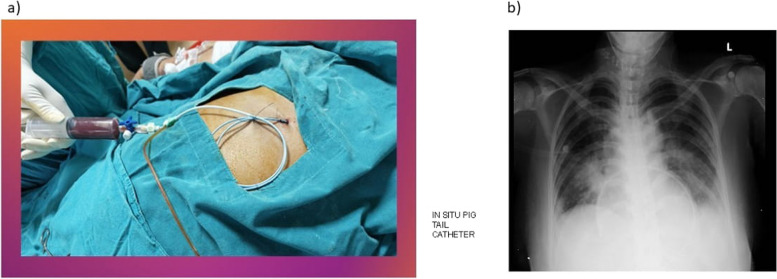
**a** Pig-tail aspiration of pericardial fluid **b** In SITU Pig tail catheter

Figure 8 shows the procedure which is used for pericardial fluid aspiration with a pig-tail catheter in the current study. The first configuration is shown in Fig. 8a, where the pig-tail catheter, under sterile conditions, is inserted into the pericardial cavity through imaging guidance like fluoroscopy or ultrasound for accuracy. Because of its coiled shape, the catheter can efficiently anchor within the pericardial cavity by preventing displacement and minimizing stress. By removing pericardial fluid gradually and carefully, this procedure lowers the risk of cardiac tamponade and relieves pressure on the heart.

As shown on a chest X-ray, Fig. 8b validates the catheter's position in situ, ensuring correct positioning for ongoing and secure draining. This approach lessens the need for frequent interventions and surgical treatments by enabling us to successfully manage fluid accumulation over time.

## Discussion

Pericardial effusion is a prevalent condition that often appears in the intensive care as well as OPDs of a tertiary care facility. The cause of PE differs throughout various regions of the globe and is associated with the varying incidence of different illnesses [12]. The symptoms of pericardial effusion differ according to rate of fluid accumulation in the pericardium, which is determined by the underlying cause. Even a small accumulation of fluid that occurs quickly may lead to tamponades, such as in situations of post-myocardial infarction and trauma. Conversely, the gradual build-up of fluid leads to symptoms within days and weeks, if a substantial quantity of fluid has collected [13]. The current study was performed among 88 patients with pericardial effusion to ascertain their etiology and the association of the etiologies with the complication of the PE in a tertiary care hospital in India.

The clinical symptoms of pericardial disorders exhibit great variation, ranging from asymptomatic to potentially endangering the life of the patients. In the index study, shortness of breath, the most common presenting complaint, was reported by 65.9% of patients, while Uddin et al. reported it among 60.6% of their patients [14]. A still higher proportion of Desale et al., Neupane et al. and Khanal et al. study patients reported shortness of breath (93.3%, 85.3% and 95%) [12, 15, 16]. In our study, fever was reported among 28.4% of the patients. A relatively lower rate of patients reported fever in Neupane et al. study (10.1%) [12]. Uddin et al. found a much higher proportion of fever among their patients (54.5%) [14]. Desale et al. and Khanal et al. also identified a higher rate of fever (50% and 47.6%) [15, 16].

Chest pain was reported by 35.2% of our patients, which is in line with the symptom pattern reported by Uddin et al. (34.8%) [14]. However, 60% of the patients in Desale et al. study presented with chest pain [16]. Neupane et al. reported chest pain only among 9% of their patients [12]. While 73.9% of the patients had raised JVP in the current study, Desale et al. and Khanal et al. study identified a relatively lower proportion of patients with raised JVP (30% and 26.9%) [15, 16]. In the present study, 50% of the patients had muffled S1 and S2, which is similar to the proportions reported in Desale et al. study (53.4%) [16]. Khanal et al. reported muffled heart sounds among 36.5% of their patients [15]. In our study, 35.2% of the patients had pulsus paradoxus, which is similar to the rates reported in Uddin et al. (33.3%) [14]. Desale et al. reported that 26.7% of their patients had pulsus paradoxus, while Khanal et al. reported it among 26.9% [15, 16]. Becks triad was seen in all the 20 patients presenting with cardiac tamponade.

Existing literature has described distinct patterns in the causes of pericardial effusion in developed nations compared to developing or under-developed countries of Asia and Africa. In the current study, it is found that the most frequent etiological factor of PE among the patients was chronic kidney disease (25%), followed by infections (22.7%)- predominantly TB (17%), and neoplastic (20.5%). The precise etiologies of uremic and dialysis-related pericarditis, which occurs in CKD, remain unclear. Uremic pericarditis, leading to PE, is believed to result from the buildup of unidentified uremic toxins, mostly because it exhibits a high resolution rate with the commencement of dialysis [17, 18]. But PE is not invariably linked to inflammation and, in a considerable proportion of chronic kidney disease patients, may be attributable to volume overload [19]. In individuals with early-stage CKD, pulmonary embolism typically originates from reasons not associated with renal pathology. Notably, among individuals with late-stage CKD, no significant correlation between serum creatinine, glomerular filtration rate, and blood urea nitrogen and the severity of PE has been reported [20, 21]. Tuberculous pericarditis (TBP) is a seldom seen variant of extrapulmonary tuberculosis, comprising 1–2% of all tuberculosis cases. TBP is the predominant etiology of significant pericardial effusion in low-income nations [22]. There is a possibility that the pericardium may be seeded in cases of military TB; nevertheless, in these cases, other organ systems will be the predominant ones. Extremely uncommon is the occurrence of direct extension from an infected visceral pleura or rib. The most common method of transmission is by the breakdown of infection in the mediastinal nodes, which then spreads straight into the pericardium, particularly in the nodes located at the tracheobronchial bifurcation [23].

Pradhan et al. reported tuberculosis, an infective cause, as the most frequent reason for pericardial effusion among their patients (94.5%) [13]. Similar to Pradhan et al., Uddin et al., Khanal et al. and Desale et al. also found TB as the major factor for PE (27.3%, 36.5% and 50%) [14–16]. Malignancy was reported relatively lower among the patients from Uddin et al. study (13.6%) [14]. Desale et al. and Khanal et al. reported neoplastic effusion among 23% and 19% of their patients, which are close to our findings [15, 16]. Effusion which is reported in malignant patients have often been due to reasons other than neoplasm itself [14]. The differential findings might be due to the relative prevalence of the conditions in the respective study areas. The methods applied to diagnose the underlying causes have also been shown to impact the distribution of etiology among pericardial effusion patients [24]. Tuberculosis is a major factor, especially in India, where it is highly endemic [25]. In developed countries, neoplasm has been attributed as the frequent etiology behind the PE [26].

While idiopathic was found as reason among 10.2% of our patients, relatively higher proportion of idiopathic cases were logged in by Uddin et al. (19.7%) [14]. However, Desale et al. and Khanal et al. reported idiopathic effusion among 10% and 12.6% of their patients, similar to our study [15, 16]. In the index study, effusions related to connective tissue diseases were seen in young individuals and were seen only in females whereas chronic kidney diseases and neoplastic etiologies were seen in older population, predominantly males.

In our study, we found that 22.7% of the patients had cardiac tamponade, similar to Singh et al. (2019) study (24.3%) [27]. In contrast, Desale et al. and Khanal et al. found that 63.3% and 63% of their patients had cardiac tamponade, either diagnosed clinically or through echocardiography [15, 16]. Gupta et al. found tamponade among 26% of their patients [28]. While 14.8% of the patients were found to have constrictive pericarditis, Neupane et al. reported 2.8% of their patients had pericarditis [12]. Malgope et al. in their study reported no incidence of constrictive pericarditis among the pediatric population who had pericardial effusion [29]. Etiology analysis of complications showed neoplastic etiologies predominantly presenting as cardiac tamponade and tuberculosis etiologies presenting as constrictive pericarditis. However, our study reported that neither the cardiac tamponade nor the constrictive pericarditis showed a significant association with etiologies of the pericardial effusion found among the patients. In contrast, Sagristà-Sauleda et al. study reported that patients who had tamponade without any features of inflammation were correlated with effusion caused by malignancy [30].

Fibrin strands and thickened fluid has been characteristic of the tuberculous PE in the echocardiography [31]. Most of the PE patients with tuberculous etiology (> 50%) in our study were found to have these features. Exudative coating exhibited a 100% sensitivity, but moderate specificity of 22%, in the diagnosis of tuberculous pericarditis, as established by Liu et al. These findings were based on the fact that thickened pericardium and fibrin threads were highly specific (94% and 88%, respectively) [31].

In the currents study, ECG was found to show low voltage complexes among most of the patients (51.1%), followed by sinus tachycardia and electrical alternans. Similarly, Neupane et al. also reported low voltage among 44% of their patients [12]. Desale et al. reported electrical alternans in 13.3% of the patients, while it was 37.6% in Neupane et al. study [12, 16]. According to the chest X-ray findings, cardiomegaly was noted among 29.5% of the cases in the index study. Khanal et al. reported cardiomegaly among vast majority of the patients (90%), in the X-ray [15].

## Limitations

The present study had the following limitations: As the research was undertaken in a single institute, external validity and generalizability of our findings are limited. Small sample size limits the internal validity of the results, which is another limitation. Long term outcomes of the patients (eg. mortality) could not be ascertained. The results are applicable only to the PE due to medical causes, which in turn limits the generalizability of the findings to the traumatic PE. Association between comorbidities/etiologies and the pericardial effusion could not be explored owing to the study design adopted- descriptive study, wherein all patients included had pericardial effusion and no control group (no-PE patients) was included. Further multi-centric, long term cohort studies must be undertaken to ascertain the validity of our findings and to improve the applicability in other settings, and to explore other complications such as mortality. Comparator group with non-PE patients needs to be added in the future studies to explore the association between comorbidities and the pericardial effusion.

## Conclusion

Overall, chronic kidney disease, closely followed by infections (mostly tuberculosis), are the frequent causes of PE in the present settings. Breathlessness is the most frequent clinical feature in the patients of PE. Fibrin strands, thickened pericardium, thickened fluid in echocardiography helps in diagnosing tubercular pericardial effusion. Cardiomegaly in chest x-ray or CT scans should further prompt towards diagnosing pericardial effusion. It is essential to incorporate these findings into the clinical practice. Patients presenting with breathlessness may be evaluated for the PE. CKD needs to be placed on par with tuberculosis while suspecting the etiology of the PE in the present settings. ADA levels in pericardial fluid (> 40 U/L) can be considered as a specific marker for tubercular PE.

## Data Availability

The datasets used and/or analysed during the current study are available from the corresponding author on reasonable request.

## Abbreviations

ADA: Adenosine Deaminase
AV: Atrioventricular
DBP: Diastolic Blood Pressure
ECHO: Echocardiography
ESR: Erythrocyte sedimentation rate
HIV: Human immunodeficiency virus
IQR: Interquartile range
IVC: Inferior vena cava
JVP: Jugular venous pressure
PE: Pericardial effusion
RA: Right atrium
RR: Respiratory Rate
SBP: Systolic Blood Pressure
SD: Standard deviation
SLE: Systemic lupus erythematosus
STEMI: ST-segment elevation myocardial infarction
TB: Tuberculosis
TSH: Thyroid stimulating hormone

## References

[1] Imazio M, Mayosi BM, Brucato A, Markel G, Trinchero R, Spodick DH, et al. Triage and management of pericardial effusion. J Cardiovasc Med (Hagerstown). 2010;11:928–35. 20814314 10.2459/JCM.0b013e32833e5788

[2] Sharma NK, Waymack JR. Acute Cardiac Tamponade. 2023. In: StatPearls [Internet]. Treasure Island (FL): StatPearls Publishing; 2024. 30521227

[3] Adler Y, Charron P, Imazio M, Badano L, Barón-Esquivias G, Bogaert J, et al. 2015 ESC Guidelines for the diagnosis and management of pericardial diseases: the task force for the diagnosis and management of pericardial diseases of the European Society of Cardiology (ESC)Endorsed by: The European Association for Cardio-Thoracic Sur. Eur Heart J. 2015;36:2921–64. 26320112 10.1093/eurheartj/ehv318 PMC7539677

[4] Azarbal A, LeWinter MM. Pericardial Effusion. Cardiol Clin. 2017;35:515–24. 29025543 10.1016/j.ccl.2017.07.005

[5] Abdel-Haq N, Moussa Z, Farhat MH, Chandrasekar L, Asmar BI. Infectious and noninfectious acute pericarditis in children: an 11-year experience. Int J Pediatr. 2018;2018:5450697. 30532791 10.1155/2018/5450697 PMC6250032

[6] Shakti D, Hehn R, Gauvreau K, Sundel RP, Newburger JW. Idiopathic pericarditis and pericardial effusion in children: contemporary epidemiology and management. J Am Heart Assoc. 2014;3:e001483. 25380671 10.1161/JAHA.114.001483 PMC4338740

[7] Peter ID, Asani MO, Aliyu I. Pericardial effusion and outcome in children at a tertiary hospital in north-western Nigeria: A 2-year retrospective review. Res Cardiovasc Med. 2019;8:14–8.

[8] Bagri NK, Yadav DK, Agarwal S, Aier T, Gupta V. Pericardial effusion in children: experience from tertiary care center in Northern India. Indian Pediatr. 2014;51:211–3. 24736909 10.1007/s13312-014-0378-z

[9] Yamani N, Abbasi A, Almas T, Mookadam F, Unzek S. Diagnosis, treatment, and management of pericardial effusion- review. Ann Med Surg. 2022;80:104142. 10.1016/j.amsu.2022.104142 PMC9283797 35846853

[10] Alerhand S, Carter JM. What echocardiographic findings suggest a pericardial effusion is causing tamponade? Am J Emerg Med. 2019;37:321–6. 30471929 10.1016/j.ajem.2018.11.004

[11] Amare S, Tadele H. Pericardial effusion in children at tertiary national referral hospital, Addis Ababa, Ethiopia: a 7-year institution based review. BMC Emerg Med. 2024;24:6. 38185639 10.1186/s12873-023-00922-7 PMC10773101

[12] Neupane KR, Simkhada R, Manandhar R, Kansakar S, Yadav D, Kadel A, et al. Clinical profile of patients admitted with pericardial effusion in Shahid Gangalal National Heart Centre, Kathmandu, Nepal. Nepal Heart J. 2023;20(1):35–8. 10.3126/njh.v20i1.55003.

[13] Pradhan A, Vishwakarma P, Bhandari M, Sethi R, Snigdha B, Narain VS, et al. Demographic, clinical and etiological profile of pericardial effusion in India: a single centre experience. Indian J Tuberc. 2022;69:220–6. 35379405 10.1016/j.ijtb.2021.08.023

[14] Uddin MJ, Singh MP, Mehdi MD. Study of etiological and clinical profile of pericardial effusion in a tertiary care hospital in Kosi region of Bihar. India Int J Adv Med. 2016;3:514–8.

[15] Khanal RR, Gajurel RM, Sahi R, Shrestha H, Poudel CM, Devkota S, et al. Study of etiological profile, clinical profile and short term outcome of patients presenting with Pericardial Effusion in a Tertiary Care Center. Nepal World J Cardiovasc Dis. 2019;9:12.

[16] Desale SS, Sharma SY, Chaudhary CK, Surnar SG. Study of etiological, and clinical profile outcome of patients, with pericardial effusion in a BKL Walawalkar Rural Medical College Dervan, Maharashtra. Int J Acad Med Pharm. 2022;4:772–6.

[17] Dad T, Sarnak MJ. Pericarditis and pericardial effusions in end-stage renal disease. Semin Dial. 2016;29:366–73. 27228946 10.1111/sdi.12517

[18] Alpert MA, Ravenscraft MD. Pericardial involvement in end-stage renal disease. Am J Med Sci. 2003;325:228–36. 12695728 10.1097/00000441-200304000-00009

[19] Eslami V, Mousavi S, Irilouzadian R, Baghsheikhi H, Fesharaki MJ, Samavat S. Pericardial effusion in patients with chronic kidney disease: a two-center study. PLoS ONE. 2024;19:e0302200. 38843270 10.1371/journal.pone.0302200 PMC11156368

[20] Ravi V, Iskander F, Saini A, Brecklin C, Doukky R. Clinical predictors and outcomes of patients with pericardial effusion in chronic kidney disease. Clin Cardiol. 2018;41:660–5. 29532495 10.1002/clc.22946 PMC6489712

[21] Chang K-W, Aisenberg GM. Pericardial effusion in patients with end-stage renal disease. Tex Heart Inst J. 2015;42:596. 26664323 10.14503/THIJ-15-5584 PMC4665297

[22] Wang S, Wang J, Liu J, Zhang Z, He J, Wang Y. A case report and review of literature: Tuberculous pericarditis with pericardial effusion as the only clinical manifestation. Front Cardiovasc Med. 2022;9:1020672. 36407454 10.3389/fcvm.2022.1020672 PMC9667942

[23] Cherian G. Diagnosis of tuberculous aetiology in pericardial effusions. Postgrad Med J. 2004;80:262–6. 15138314 10.1136/pgmj.2003.013664 PMC1742992

[24] Adlam D, Forfar JC. Pericardial disease. Medicine (Baltimore). 2014;42:660–4.

[25] Selvaraju S, Velayutham B, Rao R, Rade K, Thiruvengadam K, Asthana S, et al. Prevalence and factors associated with tuberculosis infection in India. J Infect Public Health. 2023;16:2058–65. 37948837 10.1016/j.jiph.2023.10.009

[26] Honasoge AP, Dubbs SB. Rapid Fire: Pericardial Effusion and Tamponade. Emerg Med Clin North Am. 2018;36:557–65. 30037442 10.1016/j.emc.2018.04.004

[27] Singh A, Kumar S, Himanshu D, Sethi R, Pradhan A. Clinico-epidemiological study of pericardial effusion in Northern India. Indian J Community Heal. 2019;31 3 SE-Original:322–30.

[28] Gupta M, Prasad M, Lal A, Mishra J, Ali I, Bhatta P, et al. Clinical profile and etiology of patients with pericardial effusion. J Natl Hear Lung Soc Nepal. 2023;2:97–102.

[29] Malgope R, Basu S, Sinha MK. Clinico-etiological profile of children with pericardial effusion in a tertiary care hospital in Eastern India. J Trop Pediatr. 2021;67:fmaa118. 33346812 10.1093/tropej/fmaa118

[30] Sagristà-Sauleda J, Mercé J, Permanyer-Miralda G, Soler-Soler J. Clinical clues to the causes of large pericardial effusions. Am J Med. 2000;109:95–101. 10967149 10.1016/s0002-9343(00)00459-9

[31] Liu PY, Li YH, Tsai WC, Tsai LM, Chao TH, Yung YJ, et al. Usefulness of echocardiographic intrapericardial abnormalities in the diagnosis of tuberculous pericardial effusion. Am J Cardiol. 2001;87(1133–5):A10. 11348622 10.1016/s0002-9149(01)01481-3

